# Fitness, Weight Status and Executive Functions in Adolescents: A Cluster Analysis

**DOI:** 10.1111/sms.70098

**Published:** 2025-07-28

**Authors:** Alberto Grao‐Cruces, Fátima Martín‐Acosta, Miguel Vaquero‐Solís, Abel Ruiz‐Hermosa, Daniel Camiletti‐Moirón, David Sánchez‐Oliva

**Affiliations:** ^1^ GALENO Research Group, Department of Physical Education, Faculty of Education Sciences University of Cadiz Puerto Real Spain; ^2^ Instituto de Investigación e Innovación Biomédica de Cádiz (INiBICA) Cadiz Spain; ^3^ ACAFYDE Research Group, Faculty of Sports Sciences University of Extremadura Cáceres Spain; ^4^ Social and Health Care Research Center, Universidad de Castilla‐La Mancha Cuenca Spain

**Keywords:** cognitive function, physical condition, profiles, weight status, youth

## Abstract

Low levels of physical fitness and the prevalence of obesity represent significant public health concerns that may adversely affect executive functions in adolescents. This study aimed to examine the associations between physical fitness and weight status clusters and executive functions in adolescents. A total of 1156 secondary school students aged 12–14 years participated in the study. Cluster analysis was conducted using field‐measured cardiorespiratory fitness (CRF), muscular fitness (MF), and body mass index (BMI). Core executive functions, including inhibitory control, cognitive flexibility, and working memory, were assessed using computerized tests. Five profiles were identified: (1) Thin & Unfit, (2) Normal weight & Fit, (3) Fat & Strong, (4) Fat & Unfit, and (5) Thin & Aerobic. Participants with profiles characterized by higher CRF and MF levels and lower BMI demonstrated superior executive function compared to those with unfit profiles. This study underscores the importance of maintaining adequate levels of physical fitness and appropriate weight status in adolescents to support optimal performance of core executive functions. Furthermore, it highlights the interplay of the three components in mitigating the adverse effects of deficiencies in any one component.

**Trial Registration:** Clinicaltrials.gov identifier: NCT05891054 and NCT06254638

## 1 Introduction

Childhood and adolescence are critical periods for developing both physical fitness and cognitive abilities, particularly executive functions, which are essential for academic success and lifelong mental health [1]. However, recent evidence shows concerning trends in youth populations. According to the World Health Organization [2], over 80% of adolescents globally do not meet minimum physical activity guidelines, and rates of overweight and obesity are rising sharply. In Spain, national surveys estimate that approximately 35%–40% of school‐aged children present low levels of physical fitness or excess weight, posing a serious public health concern [3].

From a physiological perspective, physical fitness, especially cardiorespiratory fitness (CRF) and muscular fitness (MF), supports brain structure and function through multiple mechanisms. These include increased cerebral blood flow, improved oxygen and nutrient delivery, and enhanced release of neurotrophic factors such as brain‐derived neurotrophic factor (BDNF) and insulin‐like growth factor 1 (IGF‐1). In contrast, excess adiposity is associated with chronic low‐grade inflammation and metabolic dysregulation, which may negatively affect cognitive performance [4, 5].

The interaction between fitness and fatness may therefore influence cognition in complex ways, with high fitness levels potentially offsetting the negative effects of excess weight, a concept known as the “fat but fit” paradox [6]. The interplay between physical fitness, weight status, and executive functions in school‐aged populations has garnered increasing attention in health studies. Physical fitness, as defined by Caspersen et al. [7] encompasses attributes that individuals possess or aim to achieve (e.g., cardiorespiratory and musculoskeletal fitness) and serves as a health marker for children and adolescents [8]. The cognitive benefits of adequate physical fitness have been extensively documented in studies involving healthy children [5] and adults [9]. In adolescents, although this relationship has been explored in multiple studies, findings remain heterogeneous depending on the methodologies employed, the specific fitness components analyzed, and the cognitive functions assessed. Therefore, further research is needed to establish a more comprehensive understanding of these associations. Several studies have examined the associations between CRF, MF, and cognitive performance in adolescents. For instance, Alonso‐Cabrera et al. [10] reported positive relationships between CRF and MF with working memory and inhibitory control, although their study included a relatively small sample (*n* = 81). In contrast, a larger study by Solis‐Urra et al. [11], which involved a sample of 1171 adolescents, provides additional evidence supporting these associations. Their findings reinforce the notion that both CRF and MF contribute to executive function performance, particularly in inhibitory control and cognitive flexibility. Conversely, Muntaner‐Mas et al. [12] conducted a two‐year longitudinal study among adolescents aged 12–14 years and found that MF was independently associated with inhibitory control, whereas no significant associations were observed for working memory. In contrast, Shigeta el al. [13] found in a cross‐sectional study of adolescents aged 15–17 years that CRF significantly predicted working memory and inhibitory control, whereas MF was not a significant predictor after controlling for CRF.

Regarding the impact of weight status and executive functions, several studies have been developed in childhood and adolescence [14, 15, 16]. For example, Likhitweerawong et al. [16] developed a meta‐analysis, and they found weight status to be a significant negative predictor of overall executive functioning in children and adolescents. In the same line, Sakib et al. [17] found a bidirectional and negative associations between body mass index (BMI) and waist circumference with executive function in a cohort study with 11 878 children aged 9–10 years old. Additionally, other studies found that adolescents with a normal weight status outperformed their overweight or obese peers in core executive functions, including inhibitory control, cognitive flexibility, attention, and working memory [14, 18].

In summary, although numerous studies have examined the relationships between fitness, weight status, and cognition separately, most have focused on children and adults, leaving a gap in research specific to adolescents. Additionally, few studies have adopted a person‐centered approach to explore how the combination of these variables influences cognitive performance during adolescence. Person‐centered analysis techniques, such as cluster analysis, provide a valuable tool for uncovering unknown patterns in data. These methods account for intra‐individual variation, classify groups based on response patterns to a set of observed indicators [19], and facilitate the assessment of complex interactions between variables (e.g., CRF, MF, and weight status).

To the best of our knowledge, only a few studies have investigated the cross‐sectional associations between fitness and weight status profiles and executive functions in youth, with most focusing on children. For example, Zhu et al. [20] reported that children with below‐average adiposity and high sprint performance demonstrated better executive functions, particularly in inhibitory control and memory, although the results were not statistically significant. Similarly, Martínez‐Vizcaíno et al. [21] found that children with above‐average adiposity but good cardiorespiratory fitness exhibited strong performance in executive functions, including inhibitory control, working memory, and cognitive flexibility. More recently, Espinoza‐Puelles et al. [22] extended these findings by analyzing a broader sample, confirming that adolescents with higher levels of cardiorespiratory and muscular fitness tend to exhibit superior executive function performance, particularly in tasks requiring cognitive flexibility and working memory.

A key strength and novelty of the present study is that it moves beyond the traditional four‐profile “fat but fit” paradox, allowing for a more flexible and data‐driven identification of fitness and weight status profiles. This person‐centered approach provides deeper insight into the distinct contributions of different components of physical fitness, namely, cardiorespiratory and muscular fitness, to better understand their impact on cognitive performance. Thus, the aims of this study were twofold: (1) to identify clusters of adolescents based on their levels of CRF, MF, and BMI; and (2) to analyze the associations of these resulting clusters with executive functions, including inhibitory control, cognitive flexibility, and working memory.

## 2 Methods

### 2.1 Design and Participants

Participants were enrolled in the ACTIVE CLASS [23] (protocol number NCT05891054) and MOVESCHOOL [24] (protocol number NCT06254638) studies. ACTIVE CLASS is a randomized controlled study aimed at analyzing the effects of incorporating physical activity into academic lessons on educational, psychosocial, and health outcomes. MOVESCHOOL is a quasi‐experimental study designed to examine the effects of a multicomponent school‐based intervention during the school day on educational, psychosocial, and health indicators. Data used in this study were collected from the baseline measurements of both projects during the first trimester of the 2022–2023 and 2023–2024 academic years, respectively. A total of 1156 Spanish adolescents (aged 12–14 years; 576 boys) recruited from 17 secondary schools located in the provinces of Cáceres and Cádiz participated in the current study.

The inclusion criteria for participants were: (i) enrolled in seventh or eighth grade (12–14 years old); (ii) without any physical disabilities or health issues that could restrict PA levels; and (iii) having parental or legal guardian consent to participate in the study. All participants took part voluntarily, and students provided their verbal assent before participation. Parents were informed about the study through a letter, which they were required to sign to provide written consent for their children to participate. Furthermore, school principals granted approval for the study to be conducted within their institutions. The study was approved by the Bioethics Committees of the Andalusian Government and the University of Cadiz (Cadiz, Spain), as well as the Bioethics and Biosafety Committees of the University of Extremadura (UEX) (Caceres, Spain).

### 2.2 Measures

All assessments were conducted within a regular school day. The fitness and weight status tests were completed during a Physical Education class period, lasting approximately 40–50 min. In an additional class period, students completed the cognitive function test, which lasted 15 min, followed by a questionnaire (complete version of the project), requiring approximately 35 min.

#### 2.2.1 Fitness

The tests for CRF and MF included in Ruiz et al. [25] were utilized. CRF was assessed using the 20‐m shuttle run test, which began at an initial speed of 8.5 km/h and increased by 0.5 km/h at each stage (each stage lasting 1 min). The final score was recorded as the total number of completed stages. Upper limb strength was evaluated with a digital hand dynamometer (TKK 5101 Grip D; Takey, Tokyo, Japan), adjusted according to established protocols for sex and hand size, as described by Ruiz et al. [26]. Each hand was tested twice, with the best value in kilograms recorded and the total score calculated by averaging the results. Lower limb muscular fitness was assessed using the standing long jump test, which was performed twice, with the longer of the two scores, measured in centimeters, recorded.

#### 2.2.2 Weight Status

Weight and height were measured twice, with participants wearing lightweight clothing and barefoot. An electronic scale (SECA 861 model; range: 0.05–130 kg; accuracy: 0.05 kg) was used, with participants standing still until their data were recorded by the investigator. Additionally, a telescopic stadiometer (SECA 225 model; range: 60–200 cm; accuracy: 1 mm) was employed. BMI was calculated as weight divided by height squared (kg/m^2^). Standardized BMI scores (zBMI) were subsequently calculated based on sex‐ and age‐specific reference values provided by the World Health Organization [27].

#### 2.2.3 Executive Functions

Core executive functions, including inhibitory control, cognitive flexibility, and working memory, were assessed using the NIH Examiner Battery [28]. This is a computerized protocol in which each participant is assessed individually, seated in front of a computer in a quiet environment free from distractions, and it has demonstrated adequate validity and reliability for assessing executive functions in children and adolescents [29]. Inhibitory control was measured using the flanker task [30], where participants direct their attention to a central fish in a row of five fish. They are instructed to quickly indicate the direction in which the central fish is pointing. Cognitive flexibility was assessed using the shifting task [28]. In this task, three figures are displayed on the screen: one at the top and one in each corner. The figures vary in color (red or blue) and shape (triangle or rectangle). The word “SHAPE” or “COLOR” is displayed and spoken aloud by the program. Participants are instructed to match the top figure (reference figure) with one of the corner figures, selecting as quickly as possible the correct figure based on the given cue (shape or color). Working memory was assessed using the N‐Back protocols [31]. In this task, a white square appears on the screen in a specific location, followed by a number that participants are asked to say aloud. A second white square then appears, and participants must indicate whether its location matches that of the previous square. For all three tests, an overall score was calculated automatically by the software, including both reaction time and accuracy [26].

#### 2.2.4 Sociodemographic Characteristics and Covariates

Sex, age (in months), educational institution, and socioeconomic status were evaluated. Specifically, the Spanish‐adapted version of the Family Affluence Scale (FAS III) [32] was used to assess socioeconomic status. This scale comprises six questions scored on a categorical scale, with the total score resulting in an aggregate index ranging from 0 to 13.

### 2.3 Data Analysis

All analyses were conducted using the JASP Statistics software package version 0.19 [33], with the level of significance set at *p* < 0.05. Prior to analysis, normality was assessed using the Kolmogorov–Smirnov test and visual inspection of Q–Q plots. Outliers were detected using boxplots and *z*‐scores (values > 3 SD). Missing data (< 2%) were handled using a complete‐case analysis approach to ensure data integrity. Initially, descriptive statistics (mean and standard deviation), sex differences, and Pearson correlations among the study variables were calculated.

For the primary analyses, K‐means cluster analysis was employed to generate profiles based on fitness and weight status. JASP was preferred over SPSS for this analysis, as it provides statistical outputs for profile comparisons (*R* ^2^, AIC, BIC, and silhouette quotient). Sex‐ and age‐standardized values for CRF and strength (a composite MF *z*‐score calculated as the average of the standardized scores from the standing long jump and handgrip tests) were included alongside zBMI as indicators in the cluster analysis. The optimal number of profiles was determined by evaluating several statistical indicators, the statistical adequacy of the solution, the sample size distribution, and the substantive meaning and theoretical alignment of the extracted profiles.

Chi‐squared tests were used to examine differences between clusters by sex. Additionally, to determine whether a mixed‐effects model was necessary, we calculated the intraclass correlation coefficient (ICC) for executive function measures. The ICC values were below 0.05, suggesting minimal school‐level clustering effects. Therefore, analysis of covariance (ANCOVA) with Bonferroni post hoc tests was conducted to analyze differences between the identified clusters in the three core executive functions. Sex, age, educational institution, and socioeconomic status were included as covariates, based on previous studies [14, 32]. Effect sizes were estimated using eta squared (*η* ^2^), which represents the proportion of variance explained by the independent variable. According to Cohen's guidelines [34], *η* ^2^ values of 0.01, 0.06, and 0.14 correspond to small, medium, and large effect sizes, respectively.

## 3 Results

Table 1 presents the descriptive statistics for fitness, weight status, and executive functions for the total sample, as well as by sex, along with correlations between these variables. Boys demonstrated significantly higher CRF, MF, and zBMI compared to girls (all *p* < 0.05). In terms of cognitive variables, boys scored significantly higher in inhibitory control and working memory (*p* < 0.05), whereas girls exhibited better performance in cognitive flexibility (*p* < 0.05). Correlational analyses revealed positive associations between CRF and MF with all core executive functions (*p* < 0.05), while zBMI was not significantly correlated.

**TABLE 1:** Descriptives and correlations of fitness, fatness, and executive functions.

	Total sample (*n* = 1156)	Boys (*n* = 576)	Girls (*n* = 580)	Correlations								
	(*M* ± SD)	(*M* ± SD)	(*M* ± SD)	1	2	3	4	5	6	7	8	9
1. Age (months)	156.12 ± 9.18	156.04 ± 9.06	156.21 ± 9.29	—								
2. SES	1.59 ± 0.56	1.61 ± 0.58	1.56 ± 0.54	−0.080**	—							
3. CRF (stages)	3.73 ± 2.10	4.57 ± 2.23	2.79 ± 1.47*	0.030	0.018	—						
4. Lower strength (cm)	142 ± 27.99	152.81 ± 28.46	131.48 ± 22.75*	0.094**	0.095**	0.566**	—					
5. Upper strength (kg)	22.42 ± 5.76	23.51 ± 6.46	21.25 ± 4.53*	0.056	0.013	0.125**	0.265**	—				
6. zBMI	0.56 ± 1.21	0.63 ± 1.26	0.47 ± 1.16*	−0.039	−0.074**	−0.274**	−0.211**	0.336**	—			
7. Inhibitory control	9.05 ± 0.70	9.10 ± 0.57	9.01 ± 0.71*	0.084**	0.057	0.118**	0.164 **	0.113**	−0.032			
8. Cognitive flexibility	8.18 ± 0.69	8.14 ± 0.67	8.24 ± 0.70**	0.017	−0.013	0.158**	0.105**	0.08**	−0.023	0.167**	—	
9. Working memory	2.12 ± 0.70	2.19 ± 0.69	2.06 ± 0.71*	0.153**	−0.022	0.141**	0.179**	0.140**	−0.028	0.483**	0.205**	—

Abbreviations: CRF, cardiorespiratory fitness; *M*, mean; SD, standard deviation; SES, socioeconomic status; zBMI, standardized body mass index score (according to sex‐ and age‐specific standard values from the World Health Organization).

* *p* < 0.05.

** *p* < 0.01.

Table 2 provides information regarding the identification of profiles based on physical fitness and weight status. The table includes *R* ^2^ values, AIC, BIC, silhouette quotient, and sample size for cluster solutions ranging from 2 to 6 profiles. In general, AIC and BIC values decreased as more profiles were included in the model, indicating a consistent improvement in fit. The lowest AIC and BIC values were observed for models with 5 and 6 profiles. However, the Silhouette quotient decreased with the inclusion of additional profiles, suggesting potential misclassification of elements into less distinct groups. Regarding sample size, increasing the number of profiles resulted in less balanced distributions among the profiles. For these reasons, the 5‐profile model was chosen, as it provided the best distribution while explaining a greater percentage of variance.

**TABLE 2:** Fit indexes, entropy, and model comparisons for models from latent profile analysis.

	*R* ^2^	AIC	BIC	Silhouette	Sample size
2 Profiles	0.321	2178.530	2208.3500	0.290	567; 497
3 Profiles	0.496	1626.070	1670.800	0.290	80; 106; 69
4 Profiles	0.572	1389.330	1448.960	0.260	321; 236; 242; 265
5 Profiles	0.626	1221.960	1296.510	0.250	208; 178; 248; 214; 216
6 Profiles	0.662	113.940	1203.390	0.240	171; 217; 218; 206; 140; 112

Abbreviations: AIC, Akaike information criterion; BIC, Bayesian information criterion; *R* ^2^, explained variance for total sample.

**FIGURE 1:**
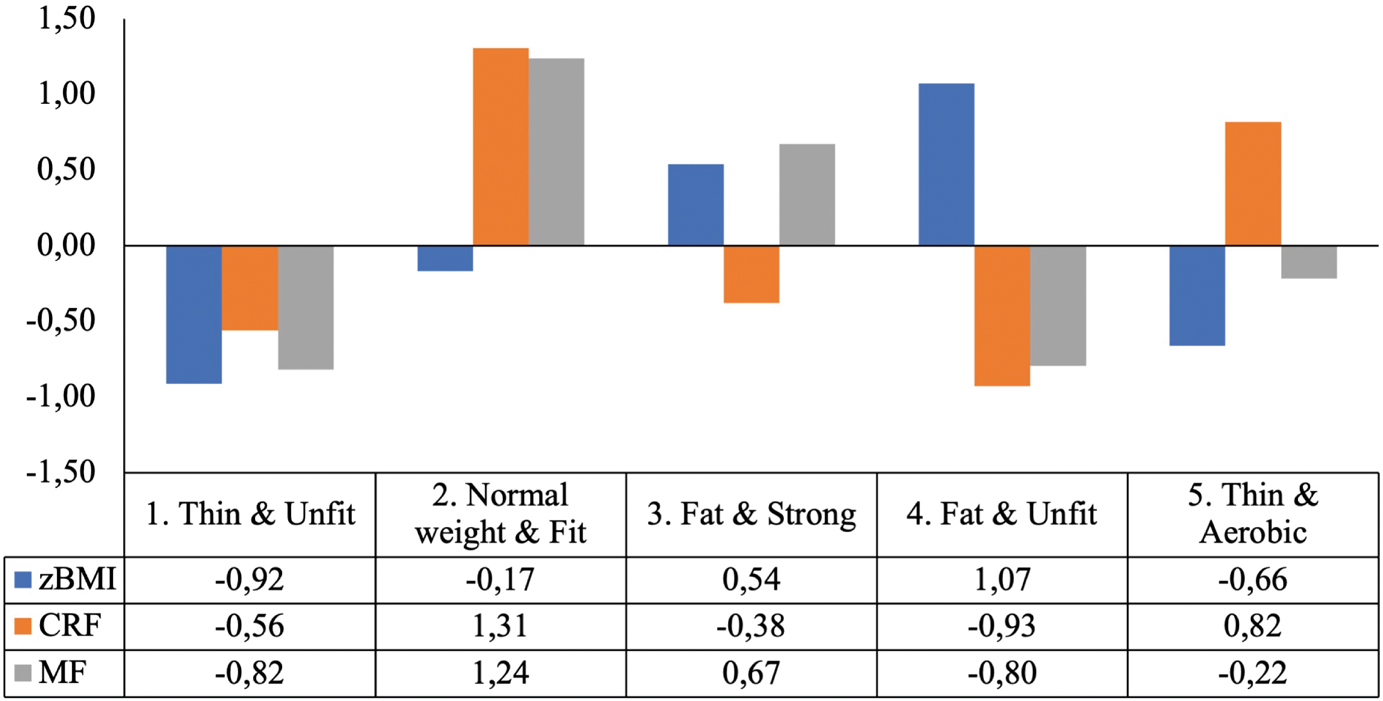
Description of the weight status and fitness variables latent profiles based on standardized scores. CRF, cardiorespiratory fitness; MF, muscular fitness; zBMI, standardized body mass index.

The characteristics of the profiles in the 5‐profile model are illustrated in Figure 1 and described in Table 3. Profile 1 (“Thin & Unfit”; *n* = 208) was characterized by moderately low scores in CRF, MF, and zBMI. Profile 2 (“Normal weight & Fit”; *n* = 178) included adolescents with high CRF and MF scores, along with average zBMI values. Profile 3 (“Fat & Strong”; *n* = 248) displayed slightly elevated zBMI, low CRF, and high MF scores. Profile 4 (“Fat & Unfit”; *n* = 214) was characterized by high zBMI alongside low CRF and MF scores. Finally, Profile 5 (“Thin & Aerobic”; *n* = 216) was characterized by slightly lower zBMI, slightly higher CRF, and average MF scores.

**TABLE 3:** Differences between profiles in fitness, fatness, and executive functions.

	1. Thin & Unfit (*n* = 208)	2. Normal weight & Fit (*n* = 178)	3. Fat & Strong (*n* = 248)	4. Fat & Unfit (*n* = 214)	5. Thin & Aerobic (*n* = 216)	*F*	*p*	*η* ^2^	Post hoc (*p* < 0.05)
Fitness and fatness									
CRF (stages)	2.586	6.155	2.956	2.018	5.408	478.614	0.001	0.662	All significant (*p* < 0.05)
Lower strength (cm)	131.29	171.43	149.55	120.01	147.40	206.785	0.001	0.458	All significant (*p* < 0.05)
Upper strength (kg)	18.260	27.345	26.205	20.976	20.418	169.370	0.001	0.409	1 < 2, 3, 4, 5; 2 > 4, 5; 3 > 4, 5
zBMI	−0.528	0.376	1.196	1.874	−0.245	303.615	0.001	0.554	All significant (*p* < 0.05)
Executive function									
Inhibitory control	8.940	9.177	9.147	8.960	9.175	5.60	0.001	0.023	1 < 2, 3, 5; 4 < 2, 5
Cognitive flexibility	7.989	8.427	8.197	8.028	8.297	12.746	0.001	0.051	1 < 2, 3, 5; 4 < 2, 5; 3 < 2
Working memory	2.056	2.299	2.091	2.011	2.203	4.635	0.001	0.019	1 < 2; 4 < 2, 5

*Note:* Higher values in executive function measures indicate better performance. All the analyses were adjusted by sex, age, educational institution, and socioeconomic status.

Abbreviations: CRF, cardiorespiratory fitness; zBMI, standardized body mass index (according to sex‐ and age‐specific standard values from the World Health Organization).

Additionally, we evaluated the distribution of each profile by sex (Table S1). A contingency table (sex × cluster) revealed a higher percentage of boys in the “Normal weight & Fit,” “Fat & Unfit,” and “Thin & Aerobic” profiles, while a higher percentage of girls was observed in the “Thin & Unfit” and “Fat & Strong” profiles.

The upper section of Table 3 shows differences between clusters in non‐standardized fitness scores and standardized weight status indicators. In summary, the “Normal weight & Fit” and “Thin & Aerobic” profiles exhibited the highest CRF values. The “Normal weight & Fit” profile also had the highest lower body strength scores, while the ‘Normal weight & Fit’ and ‘Fat & Strong’ profiles shared the highest upper body strength values. Conversely, the ‘Fat & Unfit’ profile showed the highest weight status values (all pairwise comparisons are displayed in Table 3). Additionally, Table S2 show mean and standard deviation of fitness components by cluster and sex.

The differences between profiles in terms of executive functions are presented in Table 3. The ANCOVA analyses revealed significant differences between clusters in all executive function measures (Table 3). The effect sizes (*η* ^2^) for the main analyses ranged from 0.019 to 0.051, indicating small to medium effects. Post hoc tests further identified the specific group differences. Regarding inhibitory control, the “Thin & Unfit” profile exhibited significantly lower scores compared to the “Normal weight & Fit” (*p* < 0.05), “Fat & Strong” (p < 0.05), and “Thin & Aerobic” profiles (*p* < 0.01). Additionally, the “Fat & Unfit” profile demonstrated significantly lower inhibitory control than the “Normal weight & Fit” and “Thin & Aerobic” profiles (*p* < 0.01). In terms of cognitive flexibility, adolescents in the “Thin & Unfit” profile scored significantly lower than those in the “Normal weight & Fit,” “Fat & Strong,” and “Thin & Aerobic” profiles. Finally, for working memory, adolescents in the “Normal weight & Fit” profile achieved significantly higher scores compared to those in the “Thin & Unfit” and “Fat & Unfit” profiles. Similarly, the “Thin & Aerobic” profile outperformed the “Fat & Unfit” profile in working memory.

## 4 Discussion

The aim of the present study was to investigate the associations between physical fitness, weight status, and core executive functions (inhibitory control, cognitive flexibility, and working memory) using a person‐centered approach. Our findings identified five distinct profiles, which exhibited significant differences in executive functions. These results underscore the relevance of fitness and weight status in shaping cognitive performance during adolescence.

Regarding the findings derived from the person‐centered approach, five distinct profiles were identified. Direct comparisons with previous studies are challenging due to variations in the variables used as indicators (e.g., the inclusion or exclusion of CRF and speed/agility) and the methods used to measure these variables (e.g., different assessments of fatness). Nonetheless, our findings align with prior research that explored clusters based on fitness and weight status indicators, identifying three [20] and four profiles [21, 22, 35] ranged from youth with high fitness and appropriate weight status levels to those in unhealthy profiles characterized by overweight and low fitness levels.

When comparing the resulting profiles in terms of executive function, the “Normal weight & Fit” profile achieved the most optimal scores across inhibitory control, cognitive flexibility, and working memory. According to Ortega et al. [36], the ‘Normal weight & Fit’ profile comprises adolescents with, on average, high levels of CRF (boys = percentile 70–80; girls = percentile 60–70), moderate to high levels of MF (boys = percentile 60–90; girls = percentile 50–70), and normal weight status (zBMI between 0 and 1 standard deviation) [37]. This profile demonstrated significant differences compared with profiles characterized by low fitness, namely the “Thin & Unfit” and “Fat & Unfit” profiles. These results are consistent with previous cluster‐based research [20, 21, 22], which similarly found that youth grouped into profiles with the highest fitness and appropriate weight status exhibited superior cognitive performance.

The “Thin & Aerobic” profile demonstrated the second‐highest performance across all three executive functions. This profile was characterized by adolescents with normal weight, adequate to good levels of CRF (boys: percentile 60–70; girls: percentile 50–60), but poor levels of MF (boys: percentile 10–50; girls: percentile 10–40). No significant differences in executive function were observed between this profile and the “Normal weight & Fit” profile. Pairwise comparisons revealed that the most substantial differences between these profiles were in MF. Furthermore, although both groups were classified as having normal weight [37], participants in the “Thin & Aerobic” profile displayed significantly lower zBMI values (0.376 vs. −0.245) compared to those in the “Normal weight & Fit” profile. This indicates that adolescents in the “Thin & Aerobic” profile had lower MF, but also exhibited lower weight status. In other words, in the absence of high MF, having a highly favorable weight status maintained cognitive performance comparable to that of adolescents with high fitness levels but moderate weight status.

Furthermore, the “Fat & Strong” profile was characterized by overweight adolescents [37] with poor CRF levels (boys: percentile 10–30; girls: percentile 5–20), average to good upper body strength (boys: percentile 40–80; girls: percentile 40–70), and poor to average lower body strength (boys: percentile 30–60; girls: percentile 30–40). This profile exhibited satisfactory levels of executive function, achieving the third‐highest performance across all three executive functions, with no significant differences compared to the “Normal weight & Fit” or “Thin & Aerobic” profiles, except for cognitive flexibility. Moreover, adolescents in the “Fat & Strong” profile demonstrated significantly better inhibitory control and cognitive flexibility compared to the “Thin & Unfit” profile. These results are partially consistent with those found by Espinoza‐Puelles et al. [22], who found that adolescents belonging to the Fit/Fat profile scored significantly higher in inhibitory control; however, they did not find a significantly difference in cognitive flexibility. This suggests that adequate levels of MF enabled these adolescents to achieve better cognitive performance, even when compared to those with normal weight status. The key role of MF in cognitive performance was also emphasized by Muntaner‐Mas et al. [12] who found that MF was the fitness component most strongly associated with executive function, and the only component independently associated with inhibitory control.

Lastly, adolescents in the “Thin & Unfit” and “Fat & Unfit” profiles demonstrated the poorest cognitive performance. These profiles were similar in fitness levels but differed in weight status. Both were characterized by low CRF (boys: percentile 5–30; girls: percentile 5–20) and MF (boys: percentile 5–40; girls: percentile 1–30). However, the “Thin & Unfit” profile exhibited normal weight (zBMI = −0.528), whereas the “Fat & Unfit” profile displayed an average score within the overweight range, nearing obesity thresholds (zBMI = 1.874). Both the “Thin & Unfit” and “Fat & Unfit” profiles scored significantly lower across all three executive functions compared to the “Normal weight & Fit” profile, consistent with previous studies with children [20, 21]. Additionally, these profiles scored significantly lower in inhibitory control and cognitive flexibility compared to the ‘Thin & Aerobic’ profile. Finally, the ‘Fat & Unfit’ profile also exhibited significantly lower working memory performance than the ‘Thin & Aerobic’ profile. These results are partially consistent with those found by Espinoza‐Puelles et al. [22], in that they found the Fat/Unfit profile to be associated with significantly lower cognitive flexibility, but no differences were found for working memory or inhibitory control, whereas the Thin/Unfit profile only showed lower inhibitory control when comparing to the Fat/Fit profile, but no differences were found for working memory or cognitive flexibility.

In summary, our findings have three main implications. First, the results underscore the detrimental effects of poor ‘fit & fat’ profiles on executive function performance, consistent with previous studies [20, 21]. Second, they highlight the relevance of physical fitness in explaining executive function, as the two profiles with the poorest levels of physical fitness also demonstrated the lowest cognitive performance. Third, the non‐significant differences in executive function between the ‘Thin & Unfit’ and ‘Fat & Unfit’ profiles suggest that, in adolescents with very low fitness levels, having a favorable weight status is insufficient to mitigate the negative impact of poor physical fitness on cognitive performance. This aligns with Martínez‐Vizcaino et al. [21] who similarly found no significant differences between these two profiles in the three core executive functions.

In summary, our findings confirm the importance of fitness and weight status in cognitive performance, as evaluated through executive functions. But what mechanisms can explain these associations? In recent decades, several hypotheses have been proposed. One suggested mechanism for these beneficial effects is the release of neurotrophins, such as brain‐derived neurotrophic factor (BDNF) [38]. Aerobic exercise has been shown to increase BDNF levels, which in turn support neurogenesis, synaptic plasticity, and neuronal survival in brain regions involved in executive functions, such as the prefrontal cortex. Additionally, it enhances cerebral blood flow and oxygenation, creating an optimal environment for cognitive processes [4, 39]. Furthermore, MF also plays a critical role in cognitive function, since resistance training and strength‐based exercises have been associated with increased expression of BDNF, insulin‐like growth factor‐1 (IGF‐1), and vascular endothelial growth factor (VEGF), all of which contribute to neuronal growth and brain plasticity [40].

Beyond neurotrophic factors, accumulating evidence points to cellular and molecular mechanisms such as inflammatory regulation (e.g., IL‐6, TNF‐α), oxidative stress reduction, and neurotransmitter modulation as contributors to cognitive benefits [41]. Additionally, from a neuroimaging perspective, studies utilizing structural and functional magnetic resonance imaging, magnetic resonance spectroscopy, and functional near‐infrared spectroscopy have demonstrated that higher cardiorespiratory and muscular fitness levels correlate with increased gray matter volume, enhanced functional connectivity, and improved cerebral perfusion, particularly in brain regions associated with cognitive control [42, 43].

Conversely, lower weight status, particularly the reduction of visceral adiposity, may also contribute to improved cognition by mitigating the negative effects of inflammation and insulin resistance, both of which have been linked to cognitive decline [44]. Adipose tissue, especially visceral fat, produces pro‐inflammatory cytokines that can disrupt neuronal function and impair executive processes. Chronic low‐grade inflammation, often present in individuals with higher adiposity, has been shown to impair cognitive function by disrupting neural integrity and altering neurotransmitter systems [45]. Therefore, the combination of higher fitness and lower weight status may create a synergistic effect that supports optimal cognitive functioning by promoting neuroplasticity and reducing neuroinflammation.

Our study has several limitations. Firstly, it is a cross‐sectional study, which means that the causality of the observed associations cannot be established. Future longitudinal studies are needed to determine the directionality of these relationships and assess whether improvements in fitness and weight status lead to enhanced executive functions over time. Additionally, randomized controlled trials aimed at improving fitness and weight status are necessary to validate the findings presented here. Secondly, we used age‐ and sex‐adjusted BMI *z*‐scores (zBMI) due to their widespread use and standardized interpretation. However, we acknowledge that more specific indicators of central adiposity, such as waist circumference or waist‐to‐height ratio, could provide additional insight. Thirdly, the present study focuses on CRF and MF as core indicators of physical fitness, due to their strong associations with executive function and their feasibility for assessment in school‐based settings. Although CRF and MF provide robust indicators of aerobic capacity and neuromuscular strength, which are most relevant to the cognitive domains of interest, future studies could address this limitation by incorporating a comprehensive range of physical fitness indicators (CRF, MF, and motor fitness) as well as weight status indicators. Lastly, we used the global score of each task as an indicator of cognitive performance, but future studies should include some other specific indicators, such as reaction time and percentage of accuracy.

Nevertheless, the strengths of this study should also be highlighted. It is the first study conducted with such a large sample of adolescents aimed at analyzing the association between fitness and weight status and executive function from a person‐centered perspective. Furthermore, the use of cluster analysis extends the large body of existing knowledge using variable‐centered methods and has allowed us to assess how the five groups of adolescents with different combinations of fitness or weight status may differ in cognitive performance. Also, this study advances previous research by including MF as an indicator of physical fitness, rather than relying solely on CRF. Finally, another strength of this study lies in the instruments employed; instead of using self‐reported measures, physical fitness was assessed through field‐based tests, and executive functions were evaluated individually using valid computer‐based tests.

The findings of this study have important implications for physical education programmes and public health policies aimed at improving adolescent cognitive health. Given the distinct contributions of both cardiorespiratory and muscular fitness to executive functions, school‐based programmes should emphasize a balanced approach to physical activity, incorporating both aerobic exercises and strength‐based training to optimize cognitive benefits. At the policy level, promoting structured physical activity within the school environment could serve as a preventive strategy to enhance cognitive performance and overall well‐being. Public health initiatives should encourage daily movement programmes, active commuting, and extracurricular sports, ensuring that adolescents meet international physical activity recommendations [46].

## 5 Conclusion

This study concludes that CRF, MF, and BMI are important predictors of cognitive performance in adolescents. This fact highlights the need to promote healthy lifestyles in childhood and adolescence, which can lead to adequate levels of fitness and weight status, allowing them to perform better in executive functions, important variables for academic performance. Specifically, CRF is a crucial variable, as the two profiles with better cognitive performance are those with higher CRF scores. Moreover, MF also plays a relevant role, especially in overweight adolescents, as good levels of strength mitigate the negative effects of obesity. Finally, BMI also makes a significant contribution to cognitive performance, as in adolescents with low muscle strength, good weight status contributes to good cognitive performance.

## Conflicts of Interest

The authors declare no conflicts of interest.

## Supporting information


Table S1.
Table S2.

## Data Availability

The data and codes that support the findings of this study are available in the Open Science Framework (OSF) repository: https://10.17605/OSF.IO/F7Q5W
