# Supplementary material for: Fitness, Weight Status and Executive Functions in Adolescents: A Cluster Analysis
Source: Scand J Med Sci Sports. 2025 Jul 28;35(8):e70098. doi: 10.1111/sms.70098 (PMC12302938; doi:10.1111/sms.70098)
Supplement: Supplementary file 1 — Table S1. Table S2. [file SMS-35-e70098-s001.docx]

| **Supplementary Table 1.** *Profile distribution by sex.* | | | | | | |
| --- | --- | --- | --- | --- | --- | --- |
| Sex/Cluster | 1.Thin  & Unfit  (*n* = 208) | 2.Normal weight & Fit  (*n* = 178) | 3.Fat & Strong  (*n* = 248) | 4.Fat & Unfit  (*n* = 214) | 5.Thin  & Aerobic  (*n* = 216) | Total |
| Boys | 90 | 99 | 114 | 128 | 130 | 561 |
| Girls | 118 | 79 | 134 | 86 | 86 | 503 |
| TOTAL | 208 | 178 | 248 | 214 | 216 | 1064 |

| **Supplementary Table 2.** *Descriptive statistic of fitness components by cluster and sex.* | | | | |
| --- | --- | --- | --- | --- |
|  |  | Boys |  | Girls |
|  |  | M±SD |  | M±SD |
| CRF (stages) | 1. Thin & Unfit | 3.31±1.11 |  | 2.03±0.74 |
|  | 2. Normal weight & Fit | 7.30±1.40 |  | 4.82±1.28 |
|  | 3. Fat & Strong | 3.82±1.24 |  | 2.22±0.81 |
|  | 4.Fat & Unfit | 2.29±1.08 |  | 1.72±0.58 |
|  | 5. Thin & Aerobic | 6.36±1.07 |  | 4.06±1.10 |
| Upper Strength (kg) | 1. Thin & Unfit | 18.86±4.48 |  | 17.66±3.77 |
|  | 2. Normal weight & Fit | 29.28±5.70 |  | 24.58±3.57 |
|  | 3. Fat & Strong | 27.96±5.54 |  | 24.31±3.56 |
|  | 4.Fat & Unfit | 21.33±4.86 |  | 20.27±3.48 |
|  | 5. Thin & Aerobic | 21.03±4.75 |  | 19.30±3.31 |
| Lower Strength (cm) | 1. Thin & Unfit | 143.56±20.17 |  | 121.03±19.39 |
|  | 2. Normal weight & Fit | 184.25±17.54 |  | 156.86±19.48 |
|  | 3. Fat & Strong | 161.80±20.19 |  | 138.59±16.47 |
|  | 4.Fat & Unfit | 127.44±17.99 |  | 109.50±13.78 |
|  | 5. Thin & Aerobic | 155.27±21.37 |  | 134.88±15.57 |

*Note:* CRF: Cardiorespiratory fitness.
